# Bioinformatic analysis reveals the importance of epithelial-mesenchymal transition in the development of endometriosis

**DOI:** 10.1038/s41598-020-65606-9

**Published:** 2020-05-21

**Authors:** Meihong Chen, Yilu Zhou, Hong Xu, Charlotte Hill, Rob M. Ewing, Deming He, Xiaoling Zhang, Yihua Wang

**Affiliations:** 1 Department of Gynecology, Jiangxi Maternal and Child Health Hospital, Nanchang, Jiangxi China; 2 Biological Sciences, Faculty of Environmental and Life Sciences, University of Southampton, Southampton, SO17 1BJ UK; 3 Institute for Life Sciences, University of Southampton, Southampton, SO17 1BJ UK; 4 Department of Medicine, Nanchang University, Nanchang, Jiangxi China; 5 NIHR Southampton Biomedical Research Centre, University Hospital Southampton, Southampton, SO16 6YD UK

**Keywords:** Data mining, Databases, Endocrine reproductive disorders

## Abstract

**Background:** Endometriosis is a frequently occurring disease in women, which seriously affects their quality of life. However, its etiology and pathogenesis are still unclear. **Methods:** To identify key genes/pathways involved in the pathogenesis of endometriosis, we recruited 3 raw microarray datasets (GSE11691, GSE7305, and GSE12768) from Gene Expression Omnibus database (GEO), which contain endometriosis tissues and normal endometrial tissues. We then performed in-depth bioinformatic analysis to determine differentially expressed genes (DEGs), followed by gene ontology (GO), Hallmark pathway enrichment and protein-protein interaction (PPI) network analysis. The findings were further validated by immunohistochemistry (IHC) staining in endometrial tissues from endometriosis or control patients. **Results:** We identified 186 DEGs, of which 118 were up-regulated and 68 were down-regulated. The most enriched DEGs in GO functional analysis were mainly associated with cell adhesion, inflammatory response, and extracellular exosome. We found that epithelial-mesenchymal transition (EMT) ranked first in the Hallmark pathway enrichment. EMT may potentially be induced by inflammatory cytokines such as CXCL12. IHC confirmed the down-regulation of E-cadherin (*CDH1*) and up-regulation of CXCL12 in endometriosis tissues. **Conclusions:** Utilizing bioinformatics and patient samples, we provide evidence of EMT in endometriosis. Elucidating the role of EMT will improve the understanding of the molecular mechanisms involved in the development of endometriosis.

## Introduction

Endometriosis is a frequently occurring gynaecological disease characterised by chronic pelvic pain, dysmenorrhea and infertility^1^. Its prevalence is estimated to be 10–15% of reproductive age females^2^ and around to 20–48% in infertile women^3^. Despite a number of theories being suggested to describe the molecular mechanisms underlying the development of endometriosis such as: Sampson’s theory of retrograde menstruation^4^, ectopic implantation, epigenetic factors^5^, immune and inflammatory factors^6,7^, eutopic endometrial determinism^8^, and stem cell factors^9^; disease pathogenesis is still not fully understood.

At present, there have been several studies on the gene expression profiles of endometriosis^10–13^, which have identified various differentially expressed genes (DEGs) involved in the development of endometriosis. However, due to heterogeneity between each independent experiment as a result of variations in tissue or specimens and/or different data processing methods, the identification of these DEGs is inconsistent. In this study, we integrated different studies using a non-biased approach, which may resolve these problems and enable the discovery of effective and reliable molecular markers.

We downloaded 3 microarray datasets GSE11691^11^, GSE7305^12^, GSE12768^13^, from Gene Expression Omnibus database (GEO), which contain gene expression data from endometriosis tissues and normal endometrial tissues. We then performed deep bioinformatic analysis, including identifying common DEGs, gene ontology (GO), Hallmark pathway enrichment and protein-protein interaction (PPI) network analysis. The findings were further validated by immunohistochemistry (IHC) staining in endometrial tissues from endometriosis or control patients. The aim of this study was to identify common DEGs and important pathways, and to explore potential candidate biomarkers for the diagnosis and therapeutic targets in endometriosis.

## Methods

### Original data collection

We used “endometriosis” as a keyword on the Gene Expression Omnibus (GEO) database, and 3 datasets (GSE11691, GSE7305 and GSE12768) were collected. GSE11691 was in GPL96 platform, [HG-U133A] Affymetrix Human Genome U133A Array, which included 9 endometriosis and 9 normal endometrial samples (Control samples). GSE7305 was in GPL570 platform, [HG-U133_Plus_2] Affymetrix Human Genome U133 Plus 2.0 Array, which included 10 endometriosis and 10 normal endometrial samples (Control samples). GSE12768 was in GPL7304 platform, institute Cochin HG18 60mer expression array 47Kl, which included 2 endometriosis and 2 normal endometrial samples (Control samples). The platform and series matrix files were downloaded.

### Analysis for Differentially Expressed Genes (DEGs)

RStudio software (version 3.6) was used to process and standardise the files. The CEL files of three datasets were downloaded from GEO. Raw data of the Affymetrix platform were normalised by Robust Multi-array Average (RMA) function in the affy package (version 1.64.0). Multiple probes relating to the same gene were deleted and summarised as the median value for further analysis. These 3 datasets were analyzed using the limma package (version 3.40.6) in the RStudio^14^, and genes with *P* value <0.05 and Log[FoldChange] (Log[FC]) > 1 were considered as DEGs. Overlapping DEGs from three databases were screened for subsequent GO, Hallmark pathway enrichment and PPI analysis, and were displayed with Venn diagrams.

### Analysis for GO and pathway enrichment

GO Biological Processes of DEGs were analyzed through online DAVID software^15^ (version 6.8), *P* value <0.05 as the cutoff criterion was considered statistically significant. The Hallmark pathway enrichment analysis was performed in Metascape^16^. *P* value <0.05 as the cutoff criterion was considered statistically significant.

### Protein-protein interaction (PPI) network analysis

The PPI of DEGs-encoded proteins was demonstrated by STRING (version 11.0)^17^, with search limited to “Homo sapiens” and a score> 0.700 corresponding to high confidence interaction as significant. Network construction and analyses were performed by Cytoscape (version 3.7.1). In addition, the function and pathway enrichment analysis were performed for DEGs in the modules by ClueGo (version 2.5.4), *P* value <0.05 was considered to be significant.

### Clinical sample collection

From June to October 2019, laparoscopic surgeries were performed in Jiangxi Maternal and Child Health Hospital (Nanchang, China), and 6 cases were pathologically diagnosed as ovarian endometriosis. On the staging criteria of endometriosis as stipulated by American Fertility Society revised (AFS-r), all patients with endometriosis were stage IV. Eutopic endometrial tissues were collected. The average age of the patients was (32.71 ± 1.12) years. Meanwhile, 6 cases of endometrial tissue were selected from patients with benign ovarian teratoma as the control group. The average age of patients was (32.18 ± 1.22) years.

All the collected endometrial tissues were diagnosed as proliferative endometrium after pathological histological diagnosis. There was no significant difference in the age of patients in each group (*P* value> 0.05). All menstrual cycles were normal, non-pregnant or non-lactation, and no hormonal medication was taken 6 months before the operation, and no obvious medical and surgical diseases and complications were found.

This study was approved by the Ethics Committee of Jiangxi Maternal and Child Health Hospital, China (No. EC-KT-201904). All patients had signed the informed consent for the study protocol. The experimental scheme was approved by the academic committee of Jiangxi Maternal and Child Health Hospital, and the experimental methods were carried out in accordance with the guidelines of the academic committee.

### Immunohistochemistry (IHC) and image analysis

Fresh tissue specimens were taken during the operation, rinsed with physiological saline to remove blood and other impurities, fixed with 10% formaldehyde, dehydrated with conventional gradient ethanol and embedded in paraffin, continuously sliced with a paraffin microtome, and baked at 65 °C for 1 h to dewax, and removed the glass. Tablets, soak in xylene for 40 min, and soak in absolute ethanol for 20 min. Rinse once in PBS, add the configured sodium citrate solution (pure water: sodium citrate = 1000:1), and heat to boiling. Discard the sodium citrate solution after cooling, wash with PBS, and anti-CXCL12 antibody (1:200; Proteintech, Wuhan, China, 17402-1-AP) or anti-E-cadherin (*CDH1*) antibody (1:200; Proteintech, Wuhan, China, 20874-1-AP) was incubated, followed by incubation with goat anti-mouse/rabbit IgG polymer antibody. After rinsing with PBS three times, staining was visualised using the peroxide substrate solution diaminobenzidine. Counterstained by haematoxylin, the slides were dehydrated in graded alcohol and mounted.

Image-pro Plus software was used to convert the image format and the grayscale units into optical density (IOD) units. Then area, density and IOD were selected for measure according to the manufactor’s protocol.

### Statistical analysis

Student’s *t*-test was used for statistical analysis between two different groups when variables were normally distributed, which was confirmed by Q-Q plots and the Shapiro-Wilk test (SPSS 18.0, Armonk, NY, USA). *P* value <0.05 was considered statistically significant.

### Ethics approval and consent to participate

This study was approved by the Ethics Committee of Jiangxi Provincial Maternal and Child Health Hospital, China (No. EC-KT-201904). All patients have signed the informed consent for the study protocol and reserve the right to withdraw at any time.

## Results

### Identification of Differentially Expressed Genes (DEGs) using integrated bioinformatics

**Figure 1:**
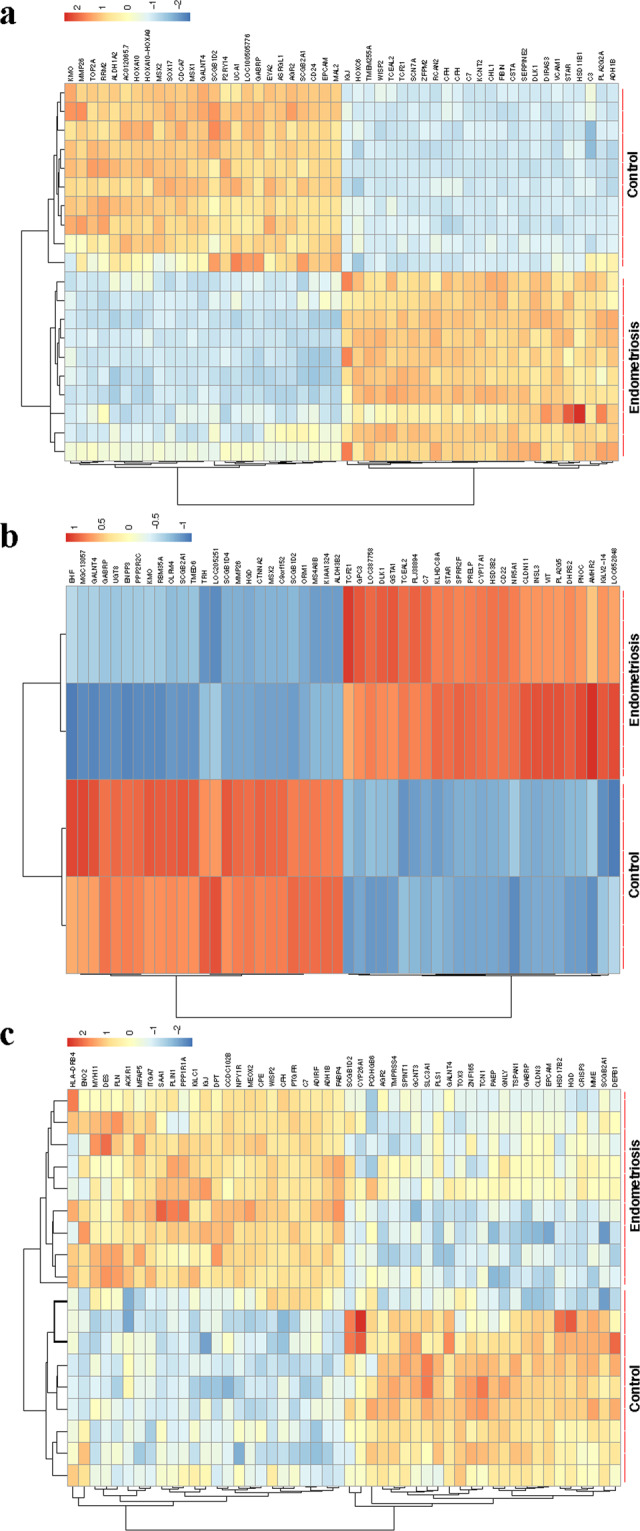
Heat maps and hierarchical clustering of the top 50 DEGs in endometriosis microarray datasets. Heat maps and hierarchal clustering analysis of top 50 DEGs in microarray datasets GSE7305 (**a**), GSE12768 (**b**), and GSE11691 (**c**). DEGs are those genes with *P* value <0.05 and Log[FC] > 1. Red indicates up-regulation and blue down-regulation.

**Figure 2:**
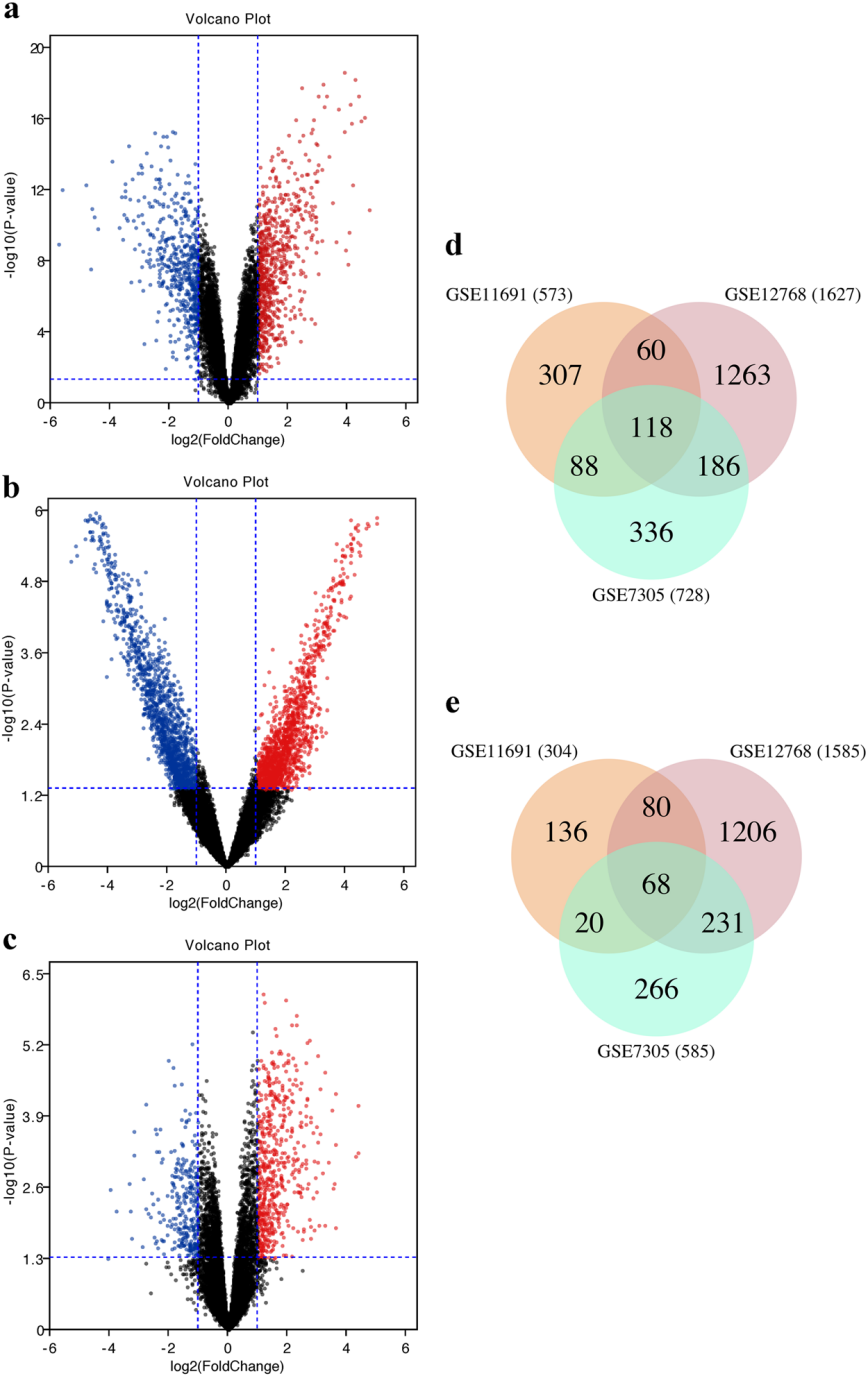
Volcano plots and Venn diagrams of DEGs in endometriosis microarray datasets. Volcano plots showing DEGs in GSE7305 (**a**), GSE12768 (**b**) and GSE11691 (**c**). DEGs are those genes with *P* value <0.05 and [logFC]> 1. Red indicates relative up-regulated genes and blue indicates down-regulated genes. Venn diagrams of up-regulated (**d**) or down-regulated (**e**) DEGs from these three datasets, as indicated.

All datasets (GSE7305, GSE11691 and GSE12768) were first normalised by Robust Multi-array Average (RMA) (Supplementary Figs. 1–3). Differential expression analysis was performed on these datasets in limma, and those genes with *P* value <0.05 and Log[FoldChange] (Log[FC]) > 1 were considered as DEGs. In GSE7305, 1,313 DEGs were identified, of which 728 genes were up-regulated and 585 down-regulated. In GSE11691, 877 DEGs were identified, with 573 up-regulated and 304 down-regulated. In GSE12768, 3,212 DEGs were identified, with 1,627 up-regulated and 1,585 down-regulated. The expression of the top 50 DEGs for all three datasets were visualised on heat maps (Fig. 1a–c). All DEGs were highlighted in Volcano plots (Fig. 2a–c). By comparing DEGs, which appeared in all 3 datasets, 186 DEGs were identified (Table 1), including 118 up-regulated (Fig. 2d) and 68 down-regulated (Fig. 2e).

**Table 1:** DEGs in endometriosis are identified by integrated bioinformatics.

DEGs	Gene Names
Up-regulated	*FMOD* | *BGN* | *CXCL12* | *MEIS2* | *ELMO1* | *AEBP1* | *MCAM* | *GPR116* | *LYZ* | *MMRN2* | *WISP* | *DPYSL3* | *ITM2A* | *NUAK1* | *TPSB2* | *COL8A2* | *CPVL* | *FMO2* | *KCTD12* | *TSPAN7* | *AQP1* | *MEOX2* | *AGTR1* | *HLA-DPB1* | *GPNMB* | *FRZB* | *FZD7* | *LY96* | *FMO1* | *PLSCR4* | *NRN1* | *CPA3* | *GAS1* | *AOC3* | *COLEC12* | *TPSAB1* | *KIAA1462* | *CPE* | *SH3BP5* | *SULF1* | *PDGFRL* | *IGJ* | *IGFBP6* | *C3* | *OLFML1* | *GLT8D2* | *CFH* | *THBS2* | *FXYD1* | *C7* | *PLP1* | *LHFP* | *ENO2* | *ITGA7* | *ACACB* | *PDLIM3* | *PRELP* | *MN1* | *FABP4* | *ROBO3* | *CSTA* | *RNASE1* | *IFI44L* | *PROS1* | *CHL1* | *VCAM1* | *VWF* | *ACTA2* | *MS4A4A* | *ARHGAP6* | *SUSD5* | *CCL* | *SELE* | *LTBP2* | *TAGLN* | *RGS2* | *SGCE* | *PTX3* | *TCF21* | *ADH1B* | *TNFSF14* | *MYH11* | *GPM6A* | *KLF2* | *GATA6* | *CNN1* | *PTPRZ1* | *CCDC69* | *CLDN5* | *TCEAL2* | *PDE2A* | *SLC16A4* | *FHL5* | *MYL9* | *GIMAP4* | *EPHA4* | *CYBRD1* | *CD163* | *FCGR2B* | *NID2* | *CFB* | *NFASC* | *HSD17B6* | *COL11A1* | *PLN* | *NTRK2* | *IGHM* | *IFIT1* | *ZFPM2* | *DES* | *ACTG2* | *ITPR1* | *CCL21* | *SCN7A* | *PLA2G2A* | *CHI3L1* | *HOXC6* | *HP*
Down-regulated	*SPINT2* | *HPN* | *GRHL2* | *ELF3* | *SH3YL1* | *TCN1* | *PPM1H* | *TSPAN1* | *ACSL5* | *PRSS16* | *BTBD3* | *TOM1L1* | *AP1M2* | *PAPSS1* | *HMGCR* | *HOXB6* | *IL20RA* | *SFN* | *EDN3* | *IRF6* | *ARG2* | *ITGB8* | *PRSS8* | *HOOK1* | *PLS1* | *PTPN3* | *PAEP* | *DEFB1* | *CLDN10* | *KIF18A* | *HSD17B2* | *SLC34A2* | *KIAA1324* | *MME* | *TPD52L1* | *GABRP* | *SLC1A1* | *ASRGL1* | *DSP* | *CDH1* | *PDZK1* | *SLC44A4* | *STX18* | *KRT19* | *DUSP4* | *DLX5* | *RAB25* | *PPAP2C* | *SALL1* | *HGD* | *PSAT1* | *PAX2* | *RORB* | *SORD* | *AGR2* | *ST14* | *TPD52* | *HOMER2* | *WFDC2* | *SLC15A2* | *CLDN3* | *GRAMD1C* | *EHF* | *CRISP3* | *PROM1* | *SLC26A2* | *CD24* | *ELP3*

### **Gene Ontology (GO) functional enrichments in DEGs**

**Figure 3:**
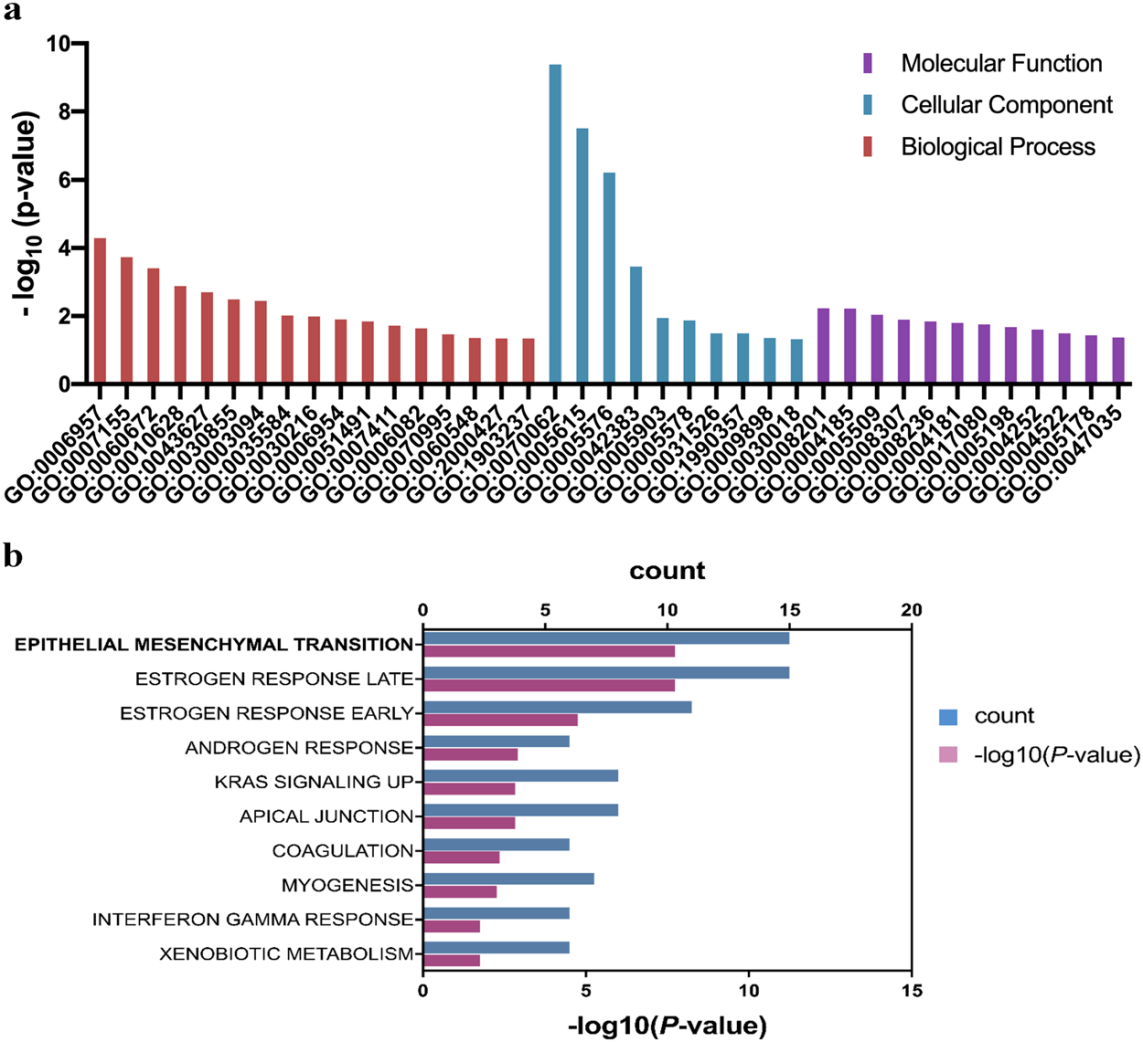
GO analysis and Hallmark pathway enrichment of DEGs in endometriosis. (**a**) GO analysis of DEGs in endometritis visualised on a bar chart clustered by molecular functions, cellular component and biological process. (**b**) Hallmark pathway enrichment of DEGs in endometriosis visualised on a bar chart, showing number of shared genes (count) and -Log_10_ (*P* value).

We then performed gene ontology (GO) enrichment analysis of DEGs in endometriosis using DAVID. The results were grouped into three categories: including molecular functions (MF), cellular component (CC) and biological process (BP) (Tables 2–4). The molecular functions of DEGs were mainly involved in calcium ion binding, heparin binding and structural molecule activity (Fig. 3a; Table 2). In the cellular component, DEGs were mainly involved in extracellular exosome, extracellular space and extracellular region (Fig. 3a; Table 3). In the biological process, DEGs were mainly involved in cell adhesion, epithelial cell differentiation, inflammatory response and extracellular exosome (Fig. 3a; Table 4).

**Table 2:** Molecular Function (MF) analysis of DEGs in endometriosis.

Term	Description	counts	*P-value (<0.05)*
GO:0008201	heparin binding	6	0.0058
GO:0004185	serine-type carboxypeptidase activity	3	0.0060
GO:0005509	calciumion binding	14	0.0091
GO:0008307	structural constituent of muscle	3	0.0126
GO:0008236	serine-type peptidase activity	3	0.0143
GO:0004181	metallocarboxypeptidase activity	3	0.0159
GO:0017080	sodium channel regulator activity	3	0.0176
GO:0005198	structural molecule activity	6	0.0210
GO:0004252	serine-type endopeptidase activity	6	0.0248
GO:0004522	ribonuclease A activity	2	0.0322
GO:0005178	integrin binding	3	0.0367
GO:0047035	testosterone dehydrogenase (NAD+) activity	2	0.0427

**Table 3:** Cellular component analysis of DEGs in endometriosis.

Term	Description	counts	*P-value (<0.05)*
GO:0070062	extracellular exosome	56	4.18E-10
GO:0005615	extracellular space	32	3.13E-08
GO:0005576	extracellular region	21	6.03E-07
GO:0042383	sarcolemma	6	3.48E-04
GO:0005903	brush border	4	0.0113
GO:0005578	proteinaceous extracellular matrix	7	0.0135
GO:0031526	brush border membrane	3	0.0321
GO:1990357	terminal web	2	0.0322
GO:0009898	cytoplasmic side of plasma membrane	3	0.0444
GO:0030018	Z disc	4	0.0482

**Table 4:** Biological process analysis of DEGs in endometriosis.

Term	Description	counts	*P-value (<0.05)*
GO:0006957	complement activation, alternative pathway	4	5.15E-05
GO:0007155	cell adhesion	10	1.84E-04
GO:0060672	epithelial cell morphogenesis involved in placenta	3	3.96E-04
GO:0010628	positive regulation of gene expression	8	0.0013
GO:0043627	response to estrogen	4	0.0019
GO:0030855	epithelial cell differentiation	5	0.0032
GO:0003094	glomerular filtration	3	0.0035
GO:0035584	calcium-mediated signaling using intracellular cal	3	0.0095
GO:0030216	keratinocyte differentiation	4	0.0102
GO:0006954	inflammatory response	8	0.0125
GO:0051491	positive regulation of filopodium assembly	3	0.0143
GO:0007411	axon guidance	5	0.0190
GO:0006082	organic acid metabolic process	2	0.0230
GO:0070995	NADPH oxidation	2	0.0343
GO:0060548	negative regulation of cell death	3	0.0441
GO:2000427	positive regulation of apoptotic cell clearance	2	0.0455
GO:1903237	negative regulation of leukocyte tethering or roll	2	0.0455

### **Signaling pathway enrichment in DEGs**

Signaling pathway enrichment of DEGs in endometriosis was performed using Metascape. The most significantly enriched pathways were submitted to Hallmark genes hit analysis. Hallmark pathway enrichment analysis identified epithelial mesenchymal transition (EMT), estrogen response late and estrogen response early as top pathways (Fig. 3b; Table 5).

**Table 5:** Hallmark pathway enrichment analysis of DEGs in endometriosis.

ID	Description	Counts	*P-value*	Gene
M5930	Epithelial mesenchymal transition	15	4.75E-11	*CXCL12* | *TAGLN* | *ACTA2* | *MYL9* | *VCAM1* | *DPYSL3* | *FMOD* | *GAS1* | *PTX3* | *ENO2* | *BGN* | *COL8A2* | *COL11A1* | *THBS2* | *NID*
M5907	Estrogen response late	15	4.75E-11	*CDH1* | *CPE* | *SLC26A2* | *SFN* | *CXCL12* | *KRT19* | *PDZK1* | *SORD* | *ST14* | *TPD52L1* | *TPSAB1* | *CCN5* | *HOMER2* | *AGR2* | *PDLIM3*
M5906	Estrogen response early	11	9.58E-09	*SLC26A2* | *ELF3* | *SFN* | *KRT19* | *PDZK1* | *CXCL12* | *SLC1A1* | *TPD52L1* | *CCN5* | *SH3BP5* | *PDLIM3*
M5908	Androgen response	6	0.0001	*SLC26A2* | *HMGCR* | *KRT19* | *SORD* | *TPD52* | *HOMER2*
M5953	Kras signaling up	8	0.0002	*CFB* | *CPE* | *CFH* | *TSPAN7* | *TSPAN1* | *GPNMB* | *BTBD3* | *LY96*
M5915	Apical junction	8	0.0002	*VWF* | *SGCE* | *MYL9* | *NFASC* | *ACTG2* | *CDH1* | *CLDN5* | *VCAM1*
M5946	Coagulation	6	0.0007	*CFB* | *C3* | *CFH* | *HPN* | *PROS1* | *VWF*
M5909	Myogenesis	7	0.0009	*AEBP1* | *DES* | *ITGA7* | *MYH11* | *FXYD1* | *TAGLN* | *TPD52L1*
M5913	Interferon gamma Response	6	0.0046	*CFB* | *CFH* | *IFIT1* | *CCL2* | *VCAM1* | *IFI44*
M5934	Xenobitoic metabolism	6	0.0046	*ARG2* | *CFB* | *FMO1* | *HSD17B2* | *PROS1* | *SPINT2*

### **Protein-protein interaction (PPI) network analysis in DEGs**

**Figure 4:**
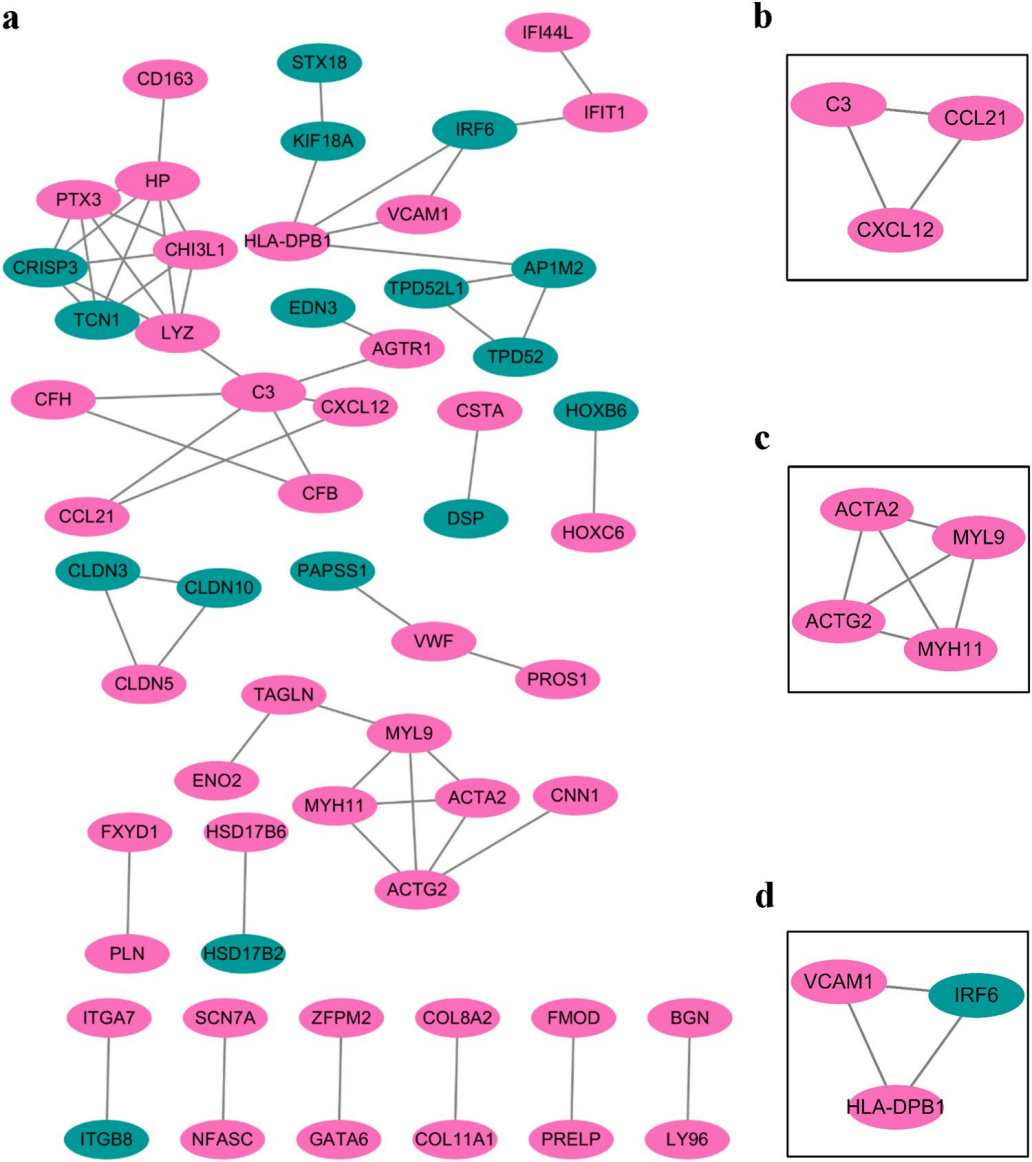
PPI network analysis of DEGs in endometriosis. Protein-Protein Interaction Network of DEGs from all datasets generated in String.db (v. 11) and visualised in Cytoscape (v. 3.7.1). (**a**) PPI network analysis of DEGs. (**b–d**) Representative local association graphs in PPI network analysis. Nodes indicate proteins/genes and lines indicate protein-protein interaction. Pink indicates up-regulation and green indicates down-regulation.

PPI analysis was performed using the online STRING database and Cytoscape software. After removing the isolated nodes and the partially connected nodes, a grid network was constructed using the Cytoscape software (Fig. 4). Pathway enrichment analysis revealed that the genes were mainly involved in vascular smooth muscle contraction, cell adhesion molecules, NF-κB pathway, complement and coagulation cascade.

### Candidate gene expression analysis and validations

**Figure 5:**
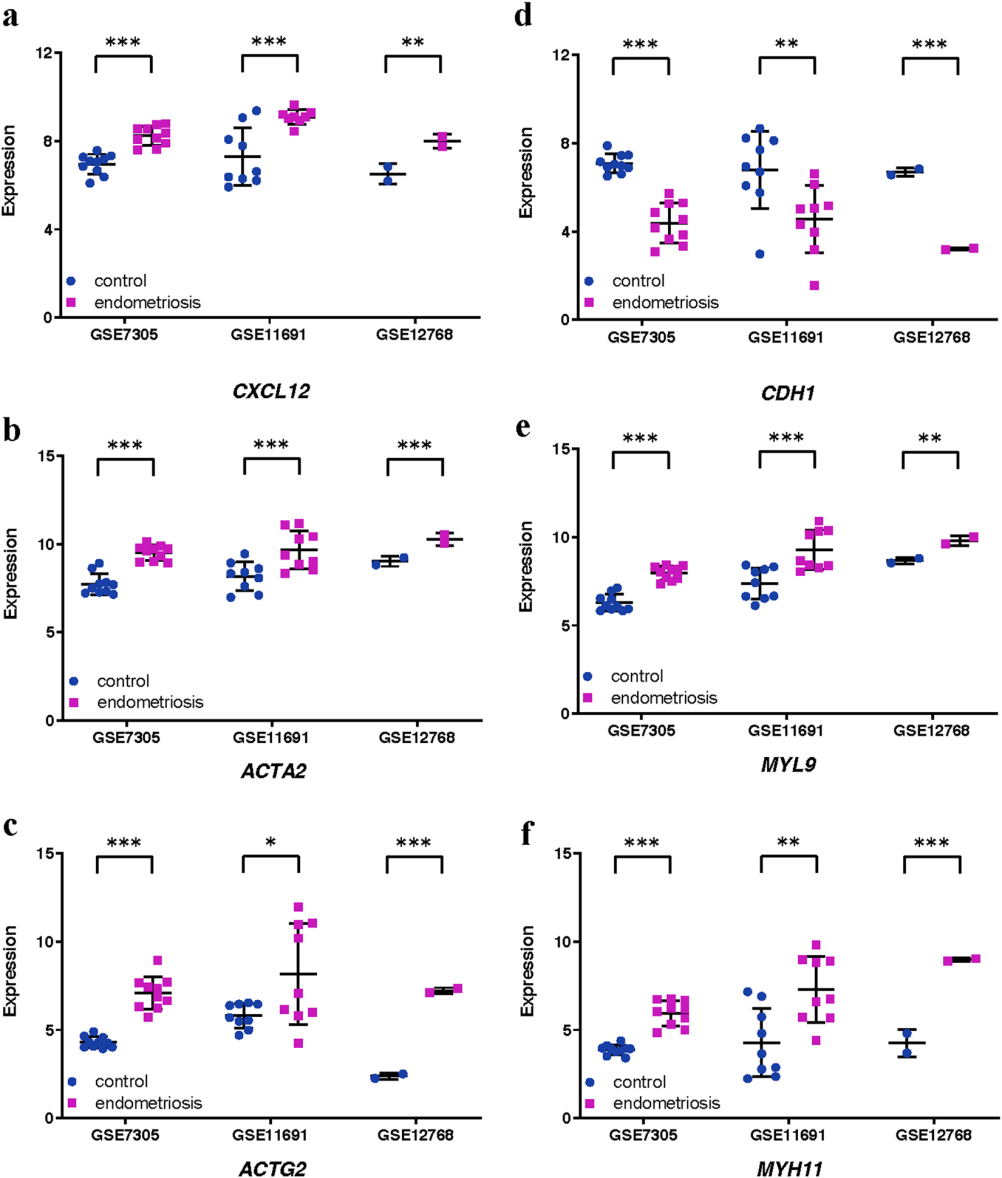
Expression levels of 6 genes in endometriosis microarray datasets. Graphs showing expression levels of *CXCL12* (**a**), *ACTA2* (**b**), *ACTG2* (**c**), *CDH1* (**d**), *MYL9* (**e**) and *MYH11* (**f**) in endometrial tissues from control (blue) or endometriosis (purple) patients in three endometriosis microarray datasets, as indicated. Data are mean ± s.d. *P value <0.05.** *P* value <0.01. *** *P* value <0.001.

**Figure 6:**
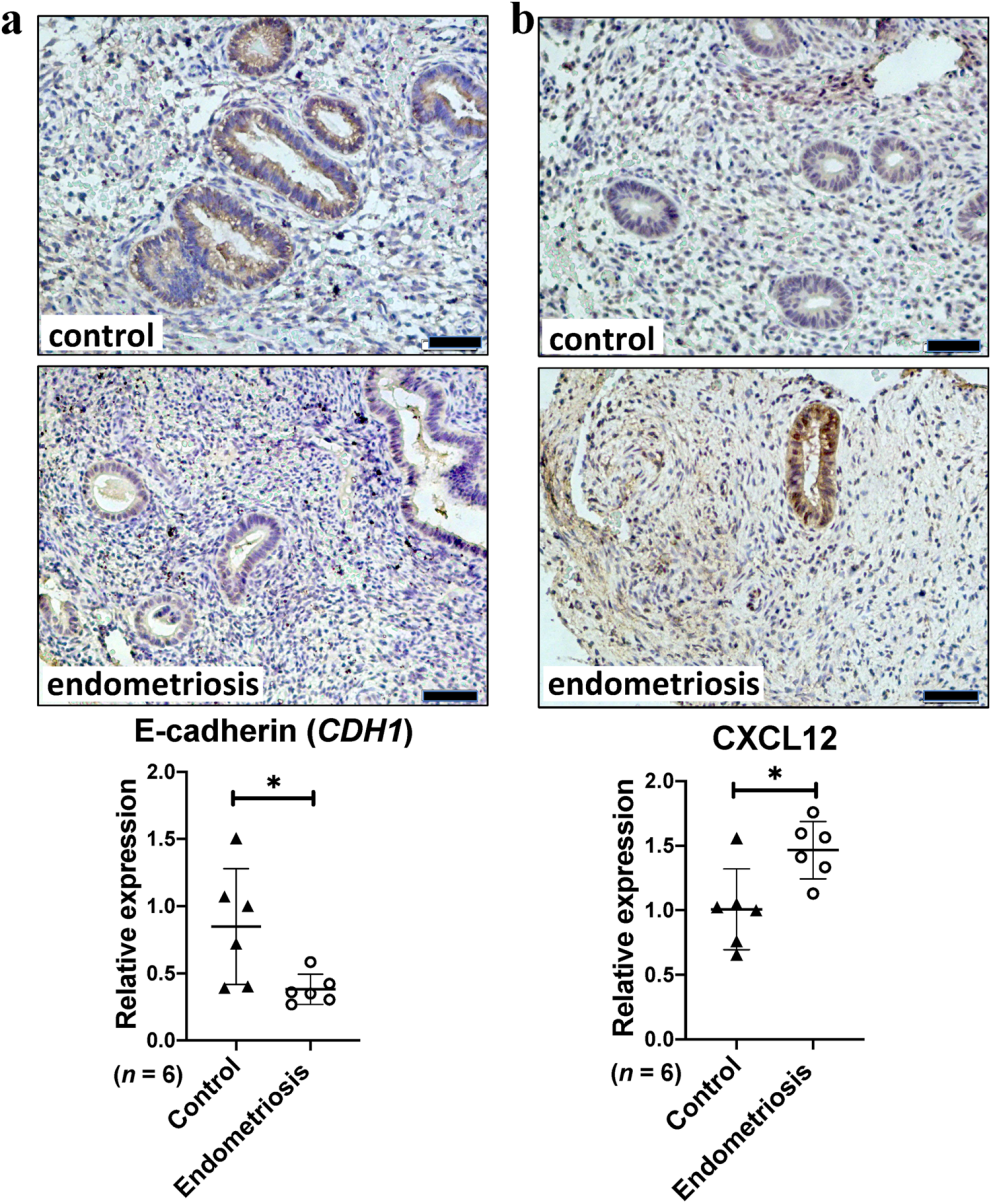
Expression levels of E-cadherin (*CDH1*) and CXCL12 in endometriosis. Representative E-cadherin (**a**) or CXCL12 (**b**) expression in endometrial tissues from control or endometriosis patients. Scale bars: 50 μm. Graphs showing comparisons of E-cadherin (**a**, *P* = 0.028) or CXCL12 (**b**, *P* = 0.015) expression in endometrial tissues from 6 control or endometriosis patients. Data are mean ± s.d.

Hallmark pathway enrichment analysis of DEGs in endometriosis identified 15 EMT-associated genes (*CXCL12*, *TAGLN*, *ACTA2*, *MYL9*, *VCAM1*, *DPYSL3*, *FMOD*, *GAS1*, *PTX3*, *ENO2*, *BGN*, *COL8A2*, *COL11A1*, *THBS2*, *NID*) (Table 5). In PPI network analysis, *CXCL12* was found to be connected to a hub gene *C3*, while *ACTG2*, *ACTA2*, *MYL9* and *MYH11* formed a connected component sub-network. In addition, a change in the expression of E-cadherin (*CDH1*) is the prototypical epithelial cell marker of EMT. As a result, although *CDH1* is not listed in Gene Set Hallmark_EMT, it was included in further analysis. Expression levels of these 6 genes (*CXCL2*, *ACTA2*, *MYL9*, *ACTG2*, *MYH11* and *CDH1*) were analysed in these three databases (Fig. 5). Significant increases were observed in *CXCL2*, *ACTA2*, *MYL9*, *ACTG2* and *MYH11* across all three databases. A significant decrease in *CDH1* was observed in all three databases. We further investigated the expression of E-cadherin (*CDH1*) and CXCL12 in endometriosis or control tissues by IHC. As shown in Fig. 6, E-cadherin was significantly down-regulated in endometriosis (Fig. 6a; *P* value = 0.028), while CXCL12 was significantly increased in endometriosis (Fig. 6b; *P* value = 0.015).

## Discussion

Endometriosis occurs in about 10–15% of reproductive age females and the etiology is unknown^1,2^. At present there is no cure and the treatment options available are limited. The disease has a high recurrence rate, which adds to its large socio-economic impact^18^. Endometriosis is the growth of cells derived from the endometrium outside the uterus, such as the ovaries, peritoneum, intestines and vagina^19^. In a small number of cases (0.5–1%) endometriosis can lead to tumor formation^20^. The underlying mechanisms of the disease are similar to malignant tumors such as cell proliferation, differentiation, apoptosis, migration, cell adhesion, invasion, and neurovascularisation^21^.

Utilising data from 3 microarray datasets (GSE11691^11^, GSE7305^12^, GSE12768^13^), we identified DEGs between endometriosis tissues and normal endometrial samples, including 118 up-regulated and 68 down-regulated genes. GO functional analysis based on these DEGs shows that DEGs are mainly enriched in cell adhesion, inflammatory response, and extracellular exosome. These findings are similar to those previously published^22^.

Importantly, Hallmark pathway enrichment analysis identified EMT as the most significant pathway. A number of studies have implicated EMT in the development of endometriosis^23–25^. EMT is a biological process where immotile epithelial cells acquire phenotypes of motile mesenchymal cells, this is accompanied by changes in cell morphology and gene expression^26^. It creates favourable conditions for the implantation and growth of endometriotic lesions^27^. During EMT the expression of a number of epithelial surface markers are lost including E-cadherin (*CDH1*), keratin, Desmoplakin, Mucin-1 and claudin; whilst a number of mesenchymal makers are up-regulated such as N-cadherin, vimentin, and fibronectin^28,29^. Numerous signaling pathways are suggested to participate in EMT induction, including transforming growth factor β (TGF-β)^30^, Wnt/β-catenin signaling pathway^31^, estrogen receptor β (ER-β)^32^, epidermal growth factor (EGF)^33^, mitogen-activated protein kinase (MAPK)/extracellular signal-regulated kinase (ERK)^34^, NF-κB^35^, estrogen receptor (ER)-α^36^ and hypoxia-inducible factor (HIF)-1α^37^. The activities of these pathways appear to be interconnected to one another, and depend on the particular epithelial or endothelial cell type affected, different signaling molecules mediate their interconnection or crosstalk. Previous studies have also found that EMT can be induced by pro-inflammatory cytokines in endometriosis, such as TGF-β^38^, tumor necrosis factor (TNF)-α^39^ and interleukin (IL)-6^40^. The mechanisms that present or activate TGF-β in the tissue microenvironment are of importance for the EMT response^41^. TGF-β induced EMT mediated by inflammatory cells in the tumor microenvironment is promoted by leukotriene B4 receptor 2, which, in response to leukotriene B4, activates reactive oxygen species (ROS) and NF-κB transcriptional activity that facilitates the establishment of EMT by TGF-β^42^.

In this unbiased study, we found EMT in endometriosis could be potentially induced by inflammatory cytokines such as C-X-C motif chemokine ligand 12 (CXCL12), also known as stromal cell-derived factor 1 (SDF1). CXCL12 is highly expressed in endometriosis in our analysis, which is consistent with a previous report^43^. CXCL12 interacts with its specific receptor, C-X-C motif chemokine receptor 4 (CXCR4), which is not consistently over-expressed in these three datasets though. The CXCL12-CXCR4 axis promotes proliferation, migration, and invasion of endometriotic cells^44,45^. In human papillary thyroid carcinoma, the CXCL12-CXCR4 axis promotes EMT processes by activating the NF-κB signaling pathway^46^. In a murine model of endometriosis both C-X-C motif chemokine receptor 7 (CXCR7) and CXCL12 expression increased with grafting time^47^. Expression of CXCR7 is enhanced during pathological inflammation and tumor development, and CXCR7 mediates TGFβ1-induced EMT^48^. However, there were no probes for *CXCR7* in the microarrays analysed in our studies. In endometriosis, it is still unclear whether CXCL12 promotes EMT through the CXCL12-CXCR4 axis or the CXCL12-CXCR7 axis. PPI analysis showed that CXCL12 interacts directly with complement C3 and C-C motif chemokine ligand 21 (CCL21), and a previous study showede CCL21 is up-regulated in endometriosis, which acts through inflammatory responses^49^. In TGF-β-induced EMT, the expression of C-C motif chemokine receptor 7 (CCR7), the CCL21 receptor, is increased and this facilitates breast cancer cell migration^50^. Through IHC, we confirmed that CXCL12 is significantly increased in endometriosis, accompanied by a decrease in the expression E-cadherin (*CDH1*), which is consistent with bioinformatics analysis. These findings, together, suggest that CXCL12 may lead to endometriosis through EMT, although further research is required.

EMT in endometriosis has been suggested to be associated with smooth muscle metaplasia and fibrogenesis^51,52^. We found various markers for smooth muscle cells in our analysis, including *ACTA2* and *MYL9*, which interact with *ACTG2* and *MYH11* in the PPI network analysis. *ACTA2* (α-SMA), is considered to be a marker of fibrosis and is up-regulated in endometriosis^53^, which is consistent with our findings. Previous studies^54,55^ have shown that platelet-derived TGF-β1 can activate the TGF-β1/Smad3 signaling pathway, subsequently promoting EMT and fibroblast-to-myofibroblast trans-differentiation (FMT) in endometriotic lesions in turn, promoting smooth muscle metaplasia and ultimately leading to fibrosis.

## Conclusion

By comparing 3 microarray datasets, we have identified 186 DEGs (118 up-regulated, 68 down-regulated) which may be involved in the progression of endometriosis. GO functional analysis determined DEGs were mainly enriched in cell adhesion, inflammatory response, and extracellular exosome. EMT was the highest ranked Hallmark pathway enrichment and we proposed that it could be induced by inflammatory cytokines and associated with smooth muscle metaplasia and fibrogenesis. Further elucidating the underlying mechanisms of endometriosis is key for the development of new treatments and bio-markers.

## Supplementary information

Supplementary information.

## Data Availability

Data and materials from this study are available upon a written request.
