# Supplementary material for: Bioinformatic analysis reveals the importance of epithelial-mesenchymal transition in the development of endometriosis
Source: Sci Rep. 2020 May 21;10:8442. doi: 10.1038/s41598-020-65606-9 (PMC7242372; doi:10.1038/s41598-020-65606-9)
Supplement: Supplementary file 1 — Supplementary information. [file 41598_2020_65606_MOESM1_ESM.pdf]

Bioinformatic analysis reveals the importance of epithelial-mesenchymal transition in the development of endometriosis

Meihong Chen[1,*], Yilu Zhou[2,3,*], Hong Xu[4], Charlotte Hill[2], Rob M. Ewing[2,3], Deming He[1], Xiaoling Zhang[1,#], Yihua Wang[2,3,5,#]

[1]Department of Gynecology, Jiangxi Maternal and Child Health Hospital, Nanchang, Jiangxi, China. [2]Biological Sciences, Faculty of Environmental and Life Sciences, University of Southampton, Southampton, SO17 1BJ, UK. [3]Institute for Life Sciences, University of Southampton, Southampton, SO17 1BJ, UK. [4] Department of Medicine, Nanchang University, Nanchang, Jiangxi, China. [5]NIHR Southampton Biomedical Research Centre, University Hospital Southampton, Southampton, SO16 6YD, UK.

[*]These authors contributed equally to this work.

[#]Correspondence should be addressed to X.Z. (e-mail: xlzzz777@126.com) or Y.W. (e-mail: yihua.wang@soton.ac.uk).

Supplementary Figure Legends

Supplementary Figure 1. Standardisation of gene expression in GSE7305 dataset. Graphs showing the data before (left) and after (right) normalisation.

Supplementary Figure 2. Standardisation of gene expression in GSE11691 dataset. Graphs showing the data before (left) and after (right) normalisation.

Supplementary Figure 3. Standardisation of gene expression in GSE12768 dataset. Graphs showing the data before (left) and after (right) normalisation.

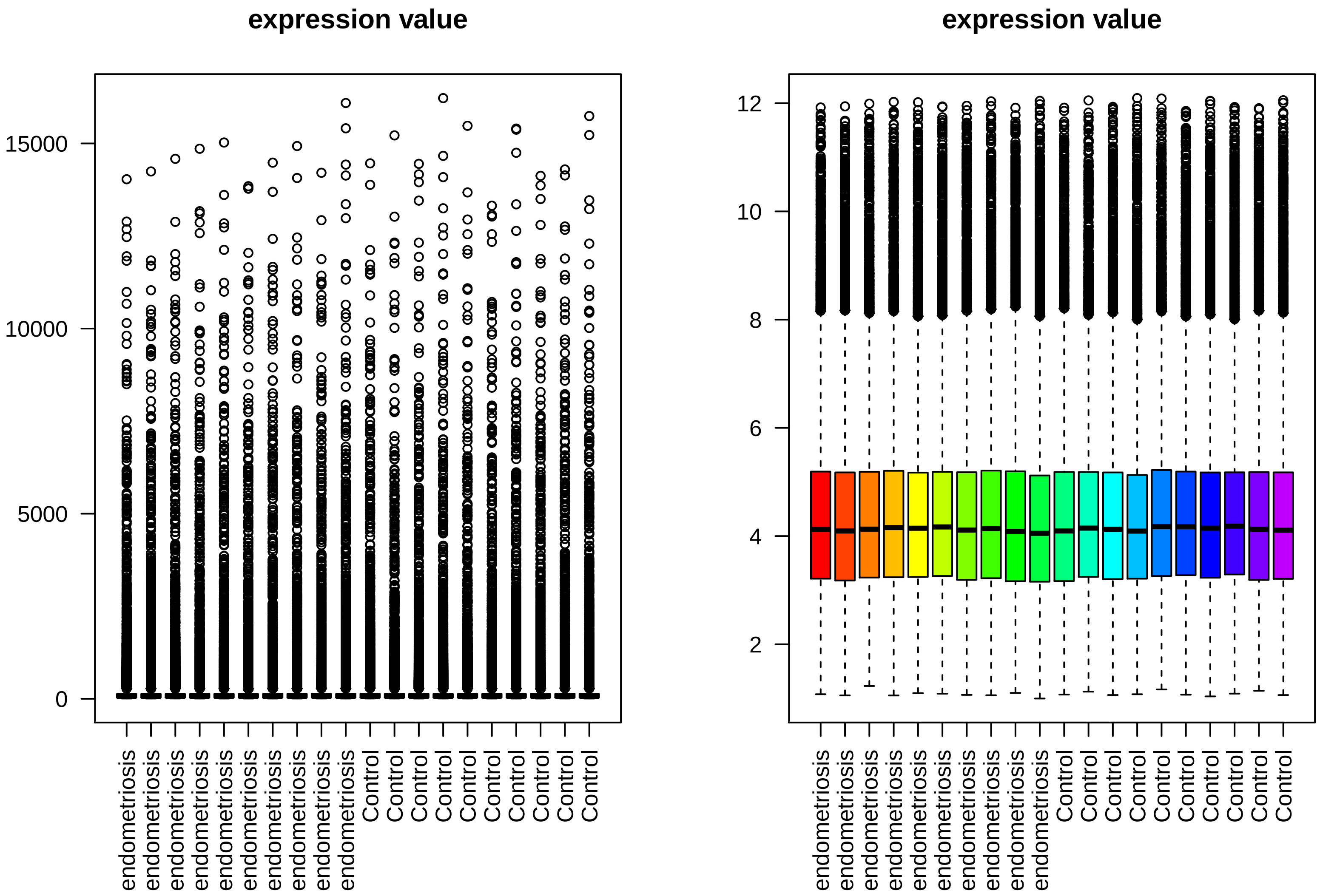

Supplementary Figure 1. Standardisation of gene expression in GSE7305 dataset.

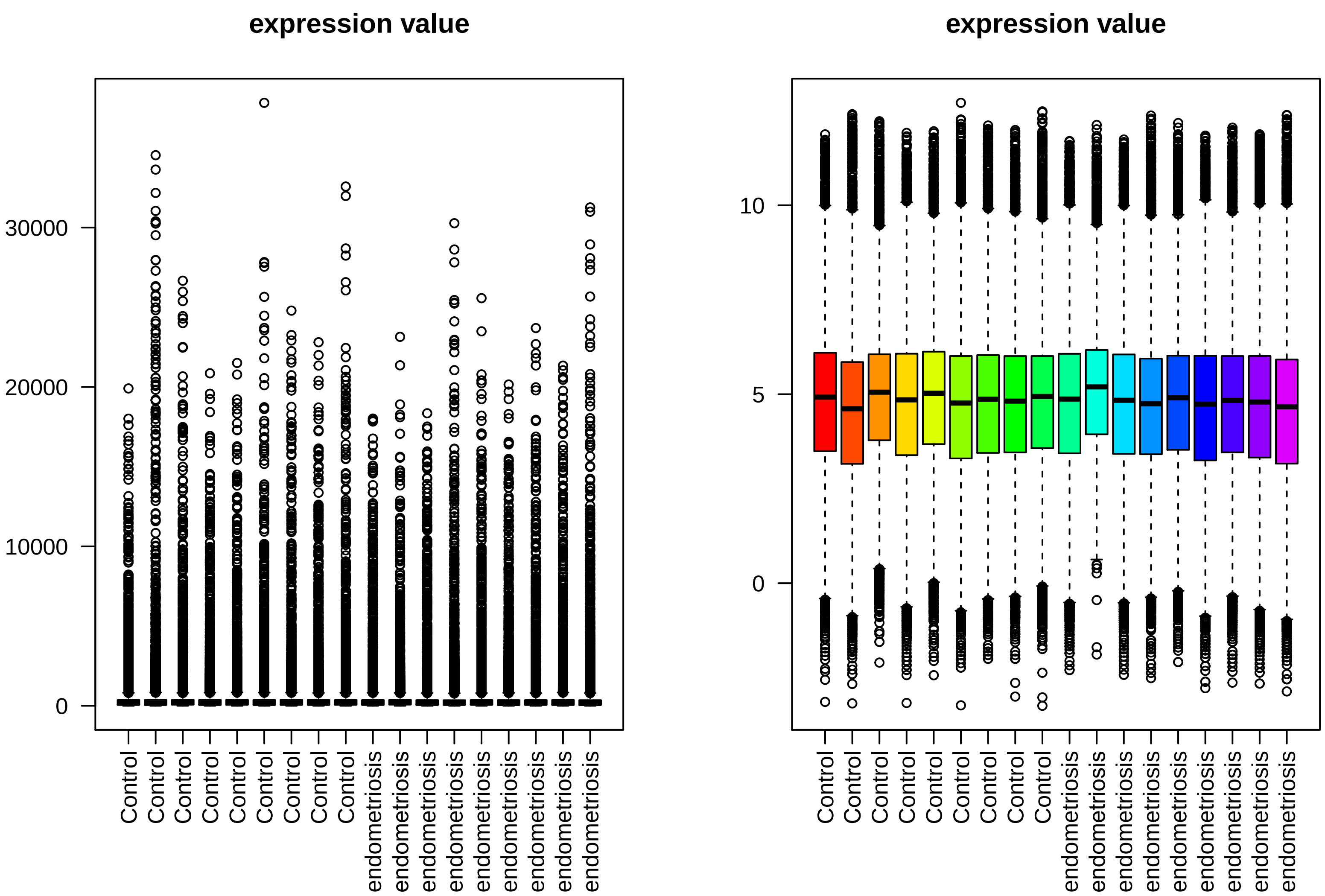

Supplementary Figure 2. Standardisation of gene expression in GSE11691 dataset.

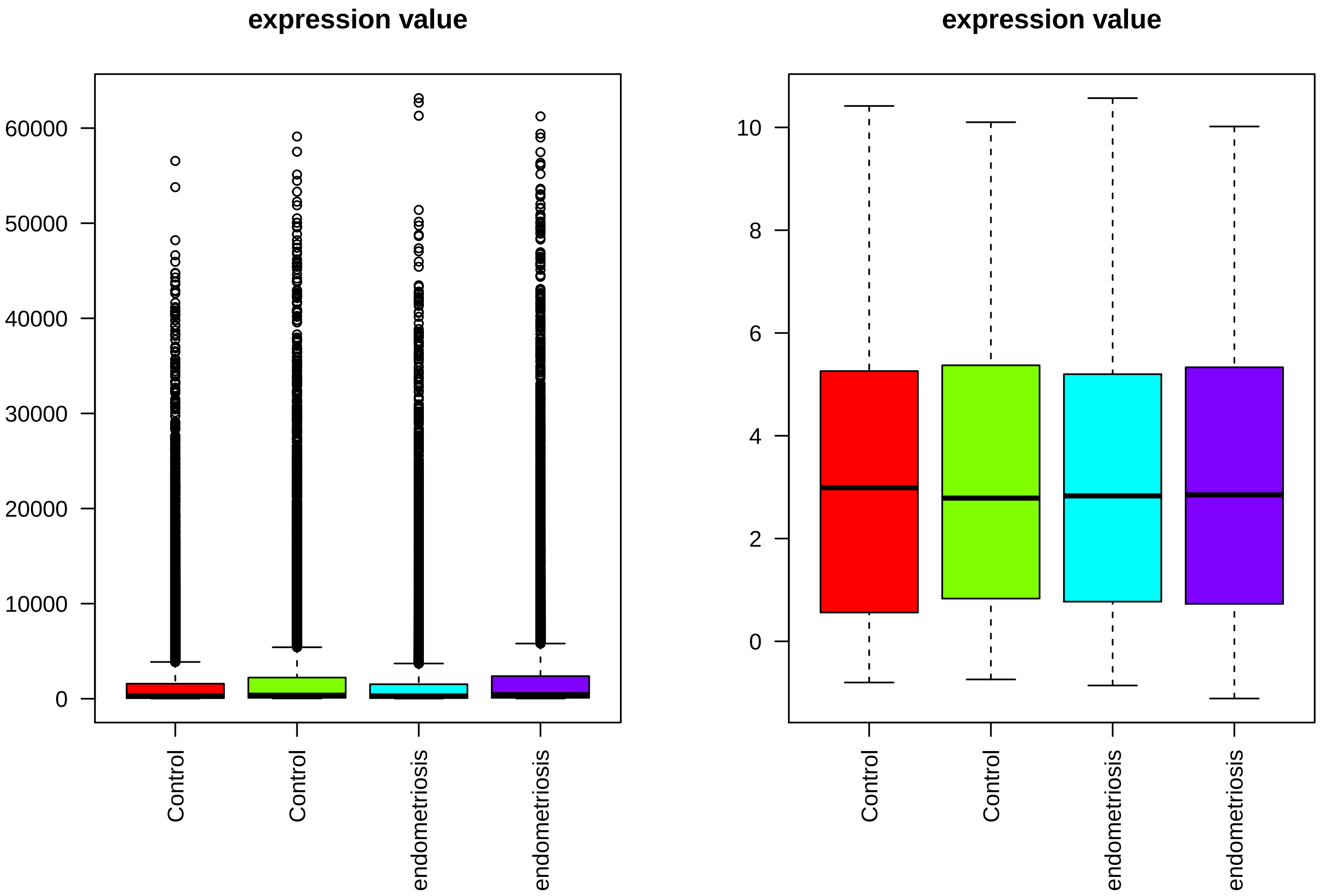

Supplementary Figure 3. Standardisation of gene expression in GSE12768 dataset.
